# The Seventh Annual Bio-Ontologies Meeting Moat House Hotel, Glasgow, 30 July 2004

**DOI:** 10.1002/cfg.433

**Published:** 2004

**Authors:** Phillip Lord, Robert Stevens

**Affiliations:** Department of Computer Science University of Manchester Kilburn Building Oxford Road Manchester M13 9PL UK

## Abstract

The Annual Bio-Ontologies Meeting [1] has now reached its seventh consecutive year, running as a special interest group (SIG) of the much larger ISMB conference. This year's meeting in Glasgow had approximately 100 attendees. Since the advent of the Gene Ontology, which coincided with the first Bio-Ontologies Meeting, we have seen a year-on-year strengthening of the field; bio-ontologies has moved from being dominated by computer science to be led by biological applications; discussion is less about ‘what is an ontology?’ and more about ‘how to build an ontology which is fit for purpose?’. This strengthening of the field can be seen elsewhere. Both the main ISMB conference and this year's Pacific Symposium on Biocomputing (PSB) [2] have seen a large number of submissions to their ontologies track. For the first time a selection of the papers from the SIG is being published in this issue of *Comparative and Functional Genomics*. We hope that this will complement the publications of the larger conferences, bringing to a wider audience the cutting edge research that characterizes the Bio-Ontologies SIG.


**Comparative and Functional Genomics**
*Comp Funct Genom* 2004; **5**: 498–500.
Published online in Wiley InterScience (www.interscience.wiley.com). **DOI:** 10.1002/cfg.433

**Conference Editorial**

### The Seventh Annual Bio-Ontologies Meeting Moat House Hotel, Glasgow, 30 July 2004

Phillip Lord* and Robert Stevens
*Department of Computer Science, University of Manchester, Kilburn Building, Oxford Road, Manchester M13 9PL, UK*

**Correspondence to:*
*Phillip Lord, Department of Computer Science, University of Manchester, Kilburn Building, Oxford Road, Manchester M13 9PL, UK.*
*E-mail: plord@cs.man.ac.uk*

Received: 1 November 2004
Revised: 2 November 2004
Accepted: 2 November 2004

### Abstract

**The Annual Bio-Ontologies Meeting [1] has now reached its seventh consecutive year, running as a special interest group (SIG) of the much larger ISMB conference. This year's meeting in Glasgow had approximately 100 attendees. Since the advent of the Gene Ontology, which coincided with the first Bio-Ontologies Meeting, we have seen a year-on-year strengthening of the field; bio-ontologies has moved from being dominated by computer science to be led by biological applications; discussion is less about 'what is an ontology?' and more about 'how to build an ontology which is fit for purpose?'. This strengthening of the field can be seen elsewhere. Both the main ISMB conference and this year's Pacific Symposium on Biocomputing (PSB) [2] have seen a large number of submissions to their ontologies track. For the first time a selection of the papers from the SIG is being published in this issue of *Comparative and Functional Genomics*. We hope that this will complement the publications of the larger conferences, bringing to a wider audience the cutting edge research that characterizes the Bio-Ontologies SIG. Copyright © 2004 John Wiley & Sons, Ltd.**

### Ontologies in bioinformatics

In the post-genomic era, biologists wish to handle data dealing with large numbers of genes, from many different species simultaneously. Often these genes and species are outside the personal knowledge of the bioinformaticians. Under these circumstances, they need to be able to gather and manipulate knowledge provided by others in an automated fashion. For this to work, a common mechanism for expressing and understanding the knowledge from different people and different sources is required. The aim, here, is not to replace the role of the biologists, but to provide a mechanism to enable them to store and organize the knowledge that they, as a community, produce.

Over the past few years the use of ontologies in enabling this automation has become commonplace, largely because of the impact of the Gene Ontology. This increasing presence of ontologies within bioinformatics has been mirrored by interest by other domains, mostly because of the increasing maturity of the Semantic Web (SW); the next generation of the Web which hopes to increase the quantity of knowledge on the web that is open to automated use and manipulation. The interaction between bioinformatics and the SW now appears to be operating to the benefit of both; the life sciences are seen by the W3C as among the early adopters of SW technology, while standards from the SW are being more heavily used in bioinformatics. This was made evident by this year's Bio-Ontologies Meeting, as several of the presentations described their use of OWL (Web Ontology Language), a new standard for representing ontologies, as well as the Resource Description Framework (RDF). It is to be hoped that increasing interest from the world at large in ontologies should result in the production of high-quality commodity software that bioinformaticians can use.

There have been a number of traditional areas for the use of ontologies, all of which were represented in the meeting. The most common use of ontologies within bioinformatics is for *description* or annotation; by attaching ontological terms to existing

Copyright © 2004 John Wiley & Sons, Ltd.

resources, we can increase the inter-resource consistency and machine understandability of these resources. A number of the presentations this year fall into this category, providing ontologies for annotations to, for example, image, anatomy and mouse phenotypes. The last subject appears as a paper in this issue by Georgios V. Gkoutos. Although less frequently seen in bioinformatics, the use of ontologies for *database management and integration* has been widely discussed. Here the ontology is used either to reconcile between different database schemas, to provide richer querying for facilities to the database, or to enable interchange between different databases. Examples of this came from a talk on PhosphaBase [3] and standardization efforts such as BioPAX [4].

The second set of talks relate to the process of producing ontologies. Improvements in *ontological engineering* have, traditionally, been the domain of the computer scientist. It was interesting to see this year that one of the talks came from the Gene Ontology Consortium and their work on OBOL [5]. The final area of interest was that of *usability*. As ontologies become more commonplace in bioinformatics, ensuring that they are simple to use becomes essential. The paper on SAEL [6] is a good example of this trend.

### The panel session

This year saw the return of a panel session to the meeting. The panelists this year were Michael Ashburner (Department of Genetics, University of Cambridge, UK), Barry Smith (IFOMIS University of Saarland and Department of Philosophy, University of Buffalo, USA), Jeremy Rogers (Department of Computer Science, University of Manchester, UK) and Crispin Miller (Paterson Institute for Cancer Research, Christie Hospital, Manchester, UK). The theme for the panel was to perform a SWOT analysis for bio-ontologies, in which the internal strengths and weaknesses of bio-ontologies are examined alongside the external opportunities and threats. As many such discussions do, this one was transformed to a discussion of the Gene Ontology — the premier example of a bio-ontology. The panel divided into two factions: the pragmatists and the philosopher. All agreed that a major strength was the very existence of GO and other ontologies and a major weakness was the risk to this existence from uncertain funding. The pragmatists simply required their ontologies to be useful, perhaps paying less attention to the philosophical niceties of distinguishing ontology from epistemology. A major schism was on the philosophical point that ontology describes reality, whereas in computer science, an ontology is a conceptualization of reality (a description of a description). This led to an extended debate on the nature of biology, bioinformatics and the modelling of those domains. Despite the potential for sparks to fly, a deal of consensus was reached on the need for high-quality ontologies and the usefulness of philosophical principles where they can be shown to be of direct impact on the utility of the ontology.

### The talks

The first talk of the day came from the keynote speaker, **David Shotton**, and the work being performed on the **BioImage** database [7]. Image databases present a particular challenge; accurate retrieval of images requires a large quantity of metadata, relating to a wide variety of information. As well as technical information about the image format, resolution and size, we need to store and search on copyright information, how the image was produced and, perhaps most importantly, information about the content, subject and interpretation of the image. While a traditional database may store the fact that the image has a panda as its main subject, information such as 'panda eating bamboo, exhibiting use of pseudo-thumb in feeding' has traditionally been stored as free text, which causes the expected difficulties in useful retrieval.

The BioImage Database has been built using SW technologies hybridised with a conventional relational database. Metadata is represented in RDF; this enables an appropriate representation for the entity–attribute–value metadata, which would only sparsely populate a relational data model. Central to the functioning of the database is the ImageOntology, written in DAML + OIL, the predecessor to OWL, which provides a central abstraction with which the web delivered front-end interacts. It is to be hoped that this work will pave the way for new and more flexible databases that biology needs for dealing with its highly complex data.

The use of an ontology as a central organizing point for a public database was also reflected in the

Copyright © 2004 John Wiley & Sons, Ltd. 

talk by **Katy Wolstencroft. PhosphaBase** contains data collated from a large number of sources describing the biology of protein phosphatases. Using the Gene Ontology to drive the automatic extraction of data from the primary data sources should ease future maintenance. This is a recurrent problem in bioinformatics, where many resources are created by small groups lacking the significant resources that such maintenance requires.

The talk by **Joanne Luciano** also related to the task of representing complex biological data. In this case, the focus is on biological pathways and enabling the interchange of this data between the existing pathway databases. **BioPAX** is an OWL ontology which has been developed for this purpose. It is currently at level 1, covering metabolic pathway data, but is expected to expand its scope. One of the key messages of the talk was the complexity of developing a standard covering even a part of bioinformatics data, and the importance of community involvement in the process. The interest in biological pathway data was maintained by **Ken-ichiro Fukuda**, who discussed their pathway database [8]. This work is described in further detail in their accompanying paper.

The expected interest in ontologies for description of biological entities continued with a number of talks. **Trish Whetzel**, described the MGED Ontology [9] that enables the description of the biological and experimental data required to interpret microarray experiments. This ontology is now relatively mature and is currently being ported to OWL; the developers wish to take advantage of the new SW facilities of the Protégé Ontology editing environment [10]. Two talks discussed applications of the ontologies to the description of anatomy. **Sarah Luger** described the application of lexical techniques to ontologies describing the anatomy of different species. This work enables the semi-automatic generation of mappings between these different ontologies, which in turn should enable cross-species queries. **Helen Parkinson's** talk described the provision of terms for broad anatomical annotation. **Keith Flanagan's** presentation covered early work on an ontology describing genomes and genomic rearrangements. These latter two are described further in their accompanying papers.

The final three talks all took a slightly different tack. **Chris Mungall's** talk about the use of lexical patterns within the Gene Ontology for error checking, and **Anand Kumar's** on the representation of granularity, are described in their papers in this issue. The day was rounded off by **Nigam Shah**, who discussed the **Hybrow** project, where an ontology has been used to describe molecular processes and their regulation in a qualitative manner. This enables the detection of contradiction and drawing of conclusions.

### Conclusions

Ontologies have now become an accepted tool of the bioinformatician. They are being used to describe many parts of biology, from anatomy, to genomics, to phenotype. With the growing maturity of Semantic Web technology, ontological tools are starting to be used as parts of data architectures. As yet, Ontologies have not become a part of the average biologists' work; this year's focus on usability and fitness for purpose suggests that this may change in the future. It is in this light that we look forward to next year's meeting.

### Acknowledgements

Phillip Lord is supported by the myGrid e-Science pilot project grant EPSRC GR/R67743.

### References

1. **http://bio-ontologies.man.ac.uk**
2. **http://psb.stanford.edu**
3. **http://www.bioinf.man.ac.uk/phosphabase/**
4. **http://www.biopax.org/**
5. **http://www.geneontology.org**
6. **http://www.iscb.org/ismb2004/posters/Duncan.DavidsonAThgu.mrc.ac.uk1_882.html**
7. **http://www.bioimage.org**
8. **http://www.inoh.org**
9. **http://mged.sourceforge.net/ontologies/index.php**
10. **http://protege.stanford.edu**

